# Human disease-related long noncoding RNAs: Impact of ginsenosides

**DOI:** 10.1016/j.jgr.2024.04.002

**Published:** 2024-04-14

**Authors:** Siyeon Jang, Hyeonjin Lee, Hyeon Woo Kim, Minjae Baek, Sanghyun Jung, Sun Jung Kim

**Affiliations:** Department of Life Science, Dongguk University-Seoul, Goyang, Republic of Korea

**Keywords:** Ginsenosides, Long noncoding RNAs, Cancer, Diagnostic markers

## Abstract

Ginsenosides in ginseng are known for their potential health benefits, including antioxidant properties and their potential to exhibit anticancer effects. Besides a various range of coding genes, ginsenosides impose their efficacy by targeting noncoding RNAs. Long noncoding RNA (

lncRNA) has gained significant attention from both basic and clinical oncology fields due to its involvement in various cancer cell activities such as proliferation, apoptosis, metastasis, and autophagy. These events can be achieved either by lncRNA alone or in association with microRNAs or proteins. This review aims to summarize the diverse activities of lncRNAs that are regulated by ginsenosides, focusing on their role in regulating target genes through signaling pathways in human diseases. We highlight the results of studies on the expression profiles of lncRNAs induced by ginsenosides in efforts to inhibit cancer cell proliferation. Finally, we discuss the potential and challenges of utilizing lncRNAs as diagnostic markers for disease treatment.

## Graphical abstract

**Figure:**
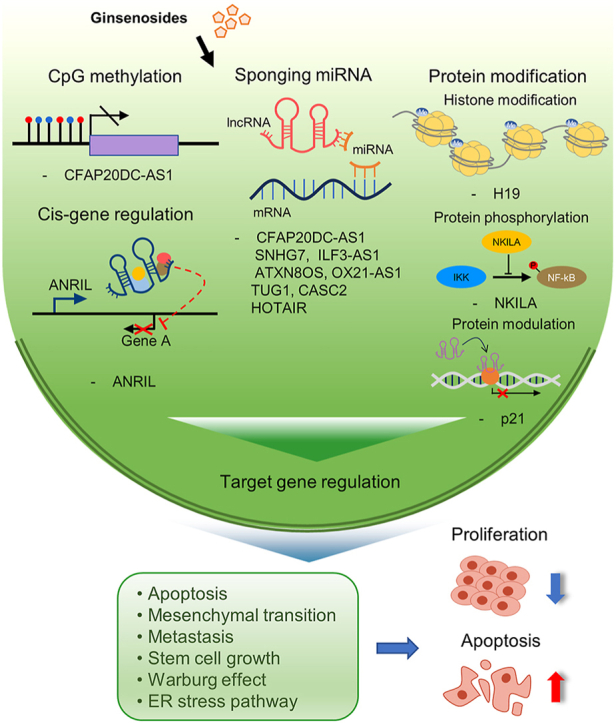

## 1 Introduction

**Fig. 1:**
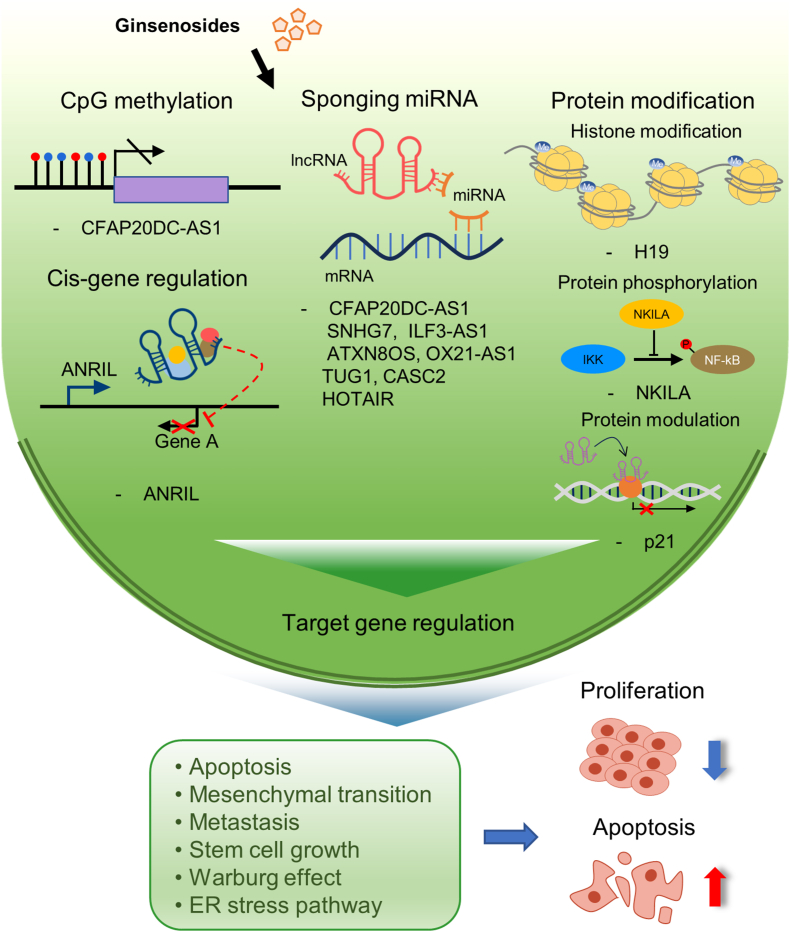
The action modes of ginsenosides and lncRNAs in cancer cells. Ginsenosides have been shown to either up- or down-regulate various lncRNAs through mechanisms that are not well elucidated. One such mechanism involves the suppression of CFAP20DC-AS1 by hypermethylation at the promoter CpGs. These lncRNAs, in turn, exert their influence through several mechanisms. They can control the expression of nearby genes (cis genes), act as sponges for microRNAs, modify histones or IκB, or modulate target proteins. These diverse events collectively regulate cancer-related target genes, resulting in the alteration of cancer cell activities such as apoptosis, metastasis, the Warburg effect, and the ER stress pathway.

Ginsenosides, which are bioactive compounds abundant in ginseng, have gained recognition for their potential health benefits, including antioxidant properties and anti-cancer effects [1,2]. These compounds can exert their efficacy by targeting noncoding RNAs, including microRNAs (miRNAs) and long non-coding RNAs (lncRNAs) [3,4]. LncRNAs, a class of noncoding RNAs longer than 200 nucleotides, do not code for proteins [5,6]. Instead, the transcripts themselves regulate the expression of other genes through diverse mechanisms [7,8]. These mechanisms include modulating the expression of nearby genes in a *cis*-acting mode [9], binding to target RNA to induce degradation or modulation [10], or regulating protein activity after forming complexes with specific proteins [11]. As of December 1st, 2023, 56,946 lncRNA genes have been identified in humans (http://lncipedia.org), with many of them being associated with cancer [12] (see Fig. 1).

Despite the prevalence of lncRNAs over miRNAs in human cells, there is limited understanding of how ginsenosides regulate lncRNAs in diseases. Further research is needed to elucidate their roles in conjunction with ginsenosides during disease progression. The complex engagement of lncRNAs in various cellular processes in cancer and other diseases, including proliferation, apoptosis, metastasis, and autophagy, has generated considerable interest in their potential as therapeutic targets [[13], [14], [15]]. This review focuses on summarizing the varied activities of lncRNAs regulated by ginsenosides, emphasizing their role in modulating target genes through signaling pathways in human diseases, particularly cancer. The exploration encompasses the expression profiles of lncRNAs induced by ginsenosides and their impact on inhibiting cancer cell proliferation. Additionally, we delve into the potential of utilizing lncRNAs as diagnostic markers for disease treatment, presenting a promising avenue for the development of targeted therapies with clinical applications [16,17].

Ginsenosides, including various types like Rh2, Rh3, Rg1, and Rg3, exhibit multifaceted pharmacological activities in numerous cancers and other diseases. For instance, Rh2 demonstrates therapeutic potential in breast cancer by intricately regulating lncRNAs such as STXBP5-AS1 [18] and CFAP20DC-AS1 (formerly known as C3orf67-AS1) [19]. Similarly, Rh3 and Rg1 exhibit diverse impacts on cancer modulation, underlining their potential in personalized therapeutic interventions [20,21]. These ginsenosides also have effects on diseases including myocardial injury, neurodegenerative disease, and skin disease [[22], [23], [24], [25]]. Moreover, other ginsenosides, including Rb3, R1, Rd, Rh7, and compound K, along with non-ginsenoside molecules in ginseng extracts, contribute to the expansive range of therapeutic effects observed [[26], [27], [28], [29], [30]]. Despite notable progress, challenges persist in understanding the complete spectrum of lncRNAs influenced by ginsenosides in cancer. Further research is warranted to unravel the intricate interactions between ginsenosides and lncRNAs, with the anticipation that novel lncRNAs will be discovered, enriching our comprehension of their roles in cancer development. This review sets the stage for future investigations into the epigenetic aspects of ginsenosides, offering valuable insights for the development of targeted cancer therapies and highlighting the molecular complexity of cancer for potential personalized therapeutic interventions.

## 2 Ginsenodides, lncRNAs, and diseases

### 2.1 Rh2 and lncRNAs

Rh2 is derived from the protopanaxadiol group, which is one of the major groups of ginsenosides [31]. Rh2 is being studied for its potential to impede cancer cell growth through multiple mechanisms. It disrupts the cell cycle, inhibiting the division of cancer cells, and induces apoptosis in specific cancer cell types [32]. Additionally, Rh2 is implicated in limiting angiogenesis, the formation of blood vessels supplying nutrients to tumors, potentially reducing the blood supply to cancer cells [33]. Moreover, it may suppress the metastatic spread of cancer cells, possibly by influencing gene expression related to metastasis [34]. The compound interacts with crucial signaling pathways like PI3K/Akt and MAPK, impacting cancer cell proliferation and survival [35].

Rh2 emerges as a pivotal player in cancer modulation by intricately regulating lncRNAs (Table 1). Specifically, Rh2 demonstrates versatile therapeutic potential in breast cancer by downregulating CFAP20DC-AS1, a miR-3614-3p sponge, thereby disrupting key pathways associated with proliferation and apoptosis. The nuanced interplay between Rh2, miR-3614-3p, and their downstream targets BBX and TNFAIP3 underscores Rh2's significance in orchestrating anti-proliferative responses in cancer cells [4]. In a separate context, Rh2 induces reversible hypermethylation of the C3orf67-AS1 promoter in MCF-7 cells, leading to its downregulation. Reduced C3orf67-AS1 inhibits cell growth, and clinical data suggests its potential role in anti-proliferative effects [19]. Furthermore, microarray analysis in HepG2 cells treated with Rh2 reveals differential expression of lncRNAs, thereby suggesting potential targets for hepatocellular carcinoma therapy [36]. This underscores the broad impact of Rh2 on cancer-related pathways. In the context of bone formation, Rh2 treatment in MC3T3-E1 cells is shown to increase the expression of lncRNA H19, leading to osteopontin (OPN) overexpression. The knockdown of H19 inhibits Rh2-induced cell proliferation and reduces OPN levels through the regulation of histone acetylation on the OPN promoter. This emphasizes the significance of H19 in Rh2-mediated bone formation [37]. Additionally, in a mouse cornea alkali burn model, Rh2 eyedrops exhibit inhibitory effects on alkali-induced neovascularization and inflammatory cell infiltrations. Notably, Rh2 attenuates the alkali-induced expression of mRNAs and lncRNAs in the cornea [38]. In a distinct context, Rh2 exhibits the ability to upregulate the expression of hub miRNAs, suppress the upregulation of prognosis-associated lncRNAs, and ultimately restore the expression of cancer-related lncRNAs to precancerous levels. Additionally, these findings position Rh2 as a potential candidate for small molecule drug development in kidney renal clear cell carcinoma (KIRC) treatment, presenting a novel approach for KIRC therapy [39].

**Table 1:** LncRNAs regulated by ginsenoside Rh2.

LncRNA	Expression alteration by ginsenoside	Mode of activity of lncRNA	Cell line/Tissue	Reference
STXBP5-AS1	Up	Sponges miR-4425 to suppress cancer cell proliferation	MCF-7/Breast	[18]
CFAP20DC-AS1	Down	Sponge miR-3614-3p to induce apoptosis	MCF-7/Breast	[4]
C3orf67-AS1	Down	Hypermethylated by Rh2 at promoter	MCF-7/Breast	[19]
Multiple lncRNAs	Variable	Potential targets for hepatoma therapy	HepG2/Liver	[36]
H19	Up	Increases osteopontin expression	MC3T3-E1	[37]
Multiple lncRNAs	Variable	Inhibit corneal neovascularization	Human umbilical vein endothelial cells	[38]
Multiple lncRNAs	Variable	Serve as biomarkers for cancer prognosis	9 types of cancer	[39]

Collectively, these diverse findings shed light on the multifaceted mechanisms by which Rh2 exerts its therapeutic effects via lncRNAs in numerous cancers, such as breast cancer and hepatocellular carcinoma, as well as in various human diseases, including bone formation and even corneal health. These insights offer hope for the advancement of interventions based on Rh2 and lncRNAs, with potential clinical implications.

### 2.2 Rg1 and lncRNAs

Rg1 exhibits diverse pharmacological activities, including anti-inflammatory effects, neuroprotection, cognitive enhancement, cardioprotection, potential anti-cancer properties, immunomodulation, and anti-fatigue effects, making it a subject of interest for its potential therapeutic applications in various health conditions [40,41]. In a study examining Müller cells under high-glucose conditions, it was revealed that Rg1 effectively mitigated mesenchymal activation and fibrosis by regulating the miR-2113/RP11-982M15.8/Zeb1 pathway. This was evidenced by its impact on MMP-2, MMP-9, and TIMP-2 expression (Table 2) [42]. Furthermore, Rg1 was found to boost proliferation and adipogenic differentiation in human adipose-derived stem cells (hASCs) by influencing pathways such as adipocytokine signaling and IL-17 signaling. This effect is mediated through regulatory factors FXR1 and Lnc-GAS5-AS1, highlighting Rg1's potential in promoting hASCs by modulating these pathways [43]. In high glucose-induced human retinal endothelial cells, Rg1 inhibited proliferation, migration, and angiogenesis by upregulating the lncRNA SNHG7. MiR-2116-5p had a target regulatory relationship with both lncRNA SNHG7 and SIRT3 [44]. Additionally, Rg1 reduced microglial activation and mitochondrial dysfunction to alleviate depression-like behavior via the gas5/ezh2/socs3/nrf2 axis [45]. In the cancer context, lncRNA GAS5 acts as a tumor suppressor by orchestrating the cell cycle, fostering apoptosis, and interacting with pivotal genes in the B-cell lymphoma development [46]. GAS5 also inhibits angiogenesis and disrupts the glucocorticoid receptor pathway, stressing its multifaceted role as a potential target for lung cancer therapies and a valuable biomarker for prognosis and diagnosis [47].

**Table 2:** LncRNAs regulated by ginsenoside Rg1.

LncRNA	Expression alteration by ginsenoside	Mode of activity of lncRNA	Cell line/Tissue	Reference
RP11-982M15.8	Down	Sponge miR-2113 to inhibit mesenchymal activation and fibrosis	MIO-M1/Retina	[42]
GAS5-AS1	Down	Suppress proliferation of adipose-derived stem cells	Human adipose-derived stem cells	[43]
SNHG7	Up	Sponge miR-2116-5p	HREC/retina	[44]
GAS5	Down	Stimulate depression-like behavior	Rat brain	[45]

### 2.3 Rg3 and lncRNAs

Rg3 demonstrates notable anti-cancer effects across various malignancies. In breast cancer, Rg3's impact intertwines with STXBP5-AS1 and RFX3-AS1, influencing cell proliferation and metastasis-free survival (Table 3) [48]. In ovarian cancer, Rg3 targets H19, disrupting the Warburg effect and tumorigenesis [49,50]. For gemcitabine-resistant pancreatic cancer, Rg3 overcomes chemoresistance by inducing apoptosis and modulating lncRNA CASC2/PTEN signaling [51]. In gliomas, Rg3 counters lncRNA NKILA-induced hypoxia, the Warburg effect, and angiogenesis [52]. In hepatocellular carcinoma, Rg3 reduces HOTAIR levels, inhibiting proliferation, migration, and invasion [53]. In osteosarcoma, Rg3 impacts SOX21-AS1, regulating mTOR and KLF4 for cell proliferation [54]. In sepsis-induced liver injury, Rg3 enhances lncRNA TUG1 expression, activating the SIRT1/AMPK pathway and promoting autophagy [55]. Additionally, Rg3 exhibits regulation of multiple lncRNAs while managing cerebral ischemia-reperfusion injury, suggesting its potential as a neuroprotectant in rats [56]. Notably, in MCF-7 breast cancer cells, Rg3-induced hypermethylation downregulates ATXN8OS, suppressing cell proliferation and promoting apoptosis [57]. In colon cancer cells, Rg3 reduces ATXN8OS, inhibiting metastasis by down-regulating miR-15b-5p and inhibiting AXIN2 [58]. Lastly, in gallbladder cancer xenografts, Rg3 inhibits tumor growth by upregulating the ER stress-mediated signaling pathway, achieved through the upregulation of the long non-coding RNA p21 [59]. These findings collectively underscore the diverse and promising anti-cancer properties of Rg3 across various cancer types, emphasizing its significant impact on lncRNA-mediated regulatory networks.

**Table 3:** LncRNAs regulated by ginsenoside Rg3.

LncRNA	Expression alteration by ginsenoside	Mode of activity of lncRNA	Cell line/Tissue	Reference
ATXN8OS	Down	Sponges miR-424-5p	MCF-7/Breast	[57]
ATXN8OS	Down	Sponges miR-15b-5p	Colon	[58]
STXBP5-AS1	Up	Increase apoptosis	MCF-7/Breast	[48]
RFX3-AS1	Down	Decrease apoptosis	MCF-7/Breast	[48]
H19	Down	Enhance Warburg effect	SKOV3/Ovary	[49]
CASC2	Up	Activate PTEN signaling	Panc-1,SW1990/Pancreas	[51]
NKILA	Down	Enhance Warburg effect	LN229 et al./Glioma	[52]
H19	Down	Enhance proliferation and invasion	SKOV3 and A2780)/Ovary	[50]
HOTAIR	Down	Enhance proliferation and invasion	SMMC-7721, SK-Hep-1/Liver	[53]
SOX21-AS1	Down	Sponge miR-7-5p, miR-145-5p	143B/Bone	[54]
TUG1	Up	Sponge miR-200a-3p	Primary hepatocytes/Liver	[55]
Multiple lncRNAs	Variable	Involved in neuroprotection	Rat brain	[56]
p21	Up	Activate ER stress pathway	GBC-SD/gall bladder	[59]

### 2.4 Other ginsenosides and lncRNAs

Ginsenosides, including Rb3, R1, Rd, Rh7, and compound K (CK), show versatile therapeutic effects. For instance, ginsenoside Rd demonstrated the suppression of SCC9 cell growth and migration, inducing apoptosis and inhibiting metastasis by downregulating lncRNA H19 and miR-675-5p, resulting in elevated CDH1 and E-cadherin expression (Table 4) [60]. In cardiac scenarios, Panax notoginseng saponins (PNS), particularly R1, enhanced cardiac function, reduced fibrosis and pyroptosis, and modulated ANRIL expression [61]. ANRIL has been implicated in various diseases, particularly cardiovascular disorders, where it modulates pathways related to inflammation, oxidative stress, and vascular dysfunction. Its dysregulation is associated with increased susceptibility to atherosclerosis, coronary artery disease, and other cardiovascular conditions [62]. PNS also exhibited anti-apoptotic effects in H9C2 cardiomyocytes during oxygen-glucose depletion (OGD)-induced myocardial ischemia, with RNA-seq analysis revealing notable changes in lncRNA expression post-PNS treatment, suggesting a potential cardioprotective role through non-coding RNA regulation [63]. Notoginsenoside R1 further lowered blood pressure in spontaneously hypertensive rats by inducing nitric oxide generation through AK094457-mediated iNOS expression [64]. Moreover, R1 demonstrated regulatory effects on proliferation, apoptosis, inflammatory response, and oxidative stress in human umbilical vein endothelial cells (HUVECs) exposed to oxidized low-density lipoprotein. This was achieved through the modulation of the XIST/miR-221-3p/TRAF6 axis via the NF-κB pathway [65].

**Table 4:** LncRNAs regulated by other ginsenosides.

LncRNA (ginsenoside)	Expression alteration by ginsenoside	Mode of activity of lncRNA	Cell line/Tissue	Reference
H19 (Rd)	Down	Enhance migration and invasion	SCC9/Tongue	[60]
ANRIL (Saponin)	Down	Enhance pyroptosis	H9C2/Rat heart	[61]
AK094457 (R1)	Down	Enhance blood pressure	WYK/Rat blood vessel	[64]
XIST (R1)	Down	Sponge miR-221-3p	Human umbilical vein endothelial cells	[65]
Multiple lncRNAs (Rf)	Variable	Inhibit tau proteotoxicity	C. elegans	[66]
Multiple lncRNAs (Saponin)	Variable	Protect cardiomyocytes from apoptosis	H9C2/Rat heart	[63]
THOR (Compound K)	Down	Enhance growth of renal carcinoma cell	Caki-1, 768-O/Kidney	[67]
H19 (Rb3)	Down	Sponge miR-29b-3p	A549, H460/Lung	[68]
ILF3-AS1 (Rh7)	Down	Sponge miR-212	A549, H1299/Lung	[29]

Ginsenoside Rf altered expression levels of lncRNAs, miRNAs, and mRNAs, potentially impacting tauopathy in neurodegenerative diseases in a worm model [66]. Eight lncRNAs (MSTRG.20812.2, MSTRG.22617.2, MSTRG.28210.13, MSTRG.5728.12, MSTRG.29708.1, MSTRG.3342.25, MSTRG.3342.31, and MSTRG.8841.8) were identified as potential targets of Rf in the inhibition of tauopathy. Ginsenoside compound K demonstrated anti-tumor effects in renal cell carcinoma by inducing apoptosis and inhibiting lncRNA THOR [67]. Additionally, Rb3 exhibited therapeutic effects in smoke-induced lung injury by modulating the H19/miR-29b-3p/HMBG1 signaling pathway [68]. Furthermore, ginsenoside Rh7, through the regulation of ILF3-AS1, influenced proliferation and metastasis in non-small cell lung cancer by modulating the miR-212/SMAD1 axis [29]. Together, these findings propose the diverse therapeutic potential of ginsenosides, emphasizing their significant impact on lncRNA-mediated regulatory networks across various physiological and pathological conditions.

### 2.5 Non-ginsenoside molecules of ginseng and lncRNAs

While this review primarily focuses on ginsenoside-regulated lncRNAs in human diseases, the inclusion of non-ginsenoside molecules from ginseng extracts broadens the scope to encompass the wider regulatory landscape. Specifically, a tRNA-derived fragment (tRF) originated from the 3′ end of tRNAGln (UUG) in ginseng directly suppresses the MIAT long noncoding RNA, resulting in increased expression of VEGFA. Introduction of a tRF mimic demonstrates notable enhancements in cardiac function, preserving cardiomyocyte cytoskeleton integrity and mitochondrial functionality [69]. In another study, a Chinese anti-tumor medicine, ADI, whose formula incorporates ginseng, was utilized to discern target genes and regulatory networks. This investigation revealed the presence of nine lncRNA nodes, including OIP5-AS1 and FDG5-AS1, within a competitive endogenous RNA regulatory network [70]. However, the specific ginseng component responsible for inducing these lncRNAs remains unidentified.

## 3 Regulatory mechanism of lncRNAs dysregulated by ginsenosides

LncRNAs play a pivotal role in cellular regulation through diverse mechanisms, encompassing sponging activity, chromatin modification, transcriptional regulation, modulation of RNA stability, influence on subcellular localization, and modulation of protein activity [71,72]. A literature review delving into the regulation of lncRNAs by ginsenosides has revealed a predominant emphasis on the modulation of protein activity and sponging activity, as outlined in the following sections. Despite significant progress, the regulatory mechanisms by which ginsenosides induce the expression of specific lncRNAs remain incompletely understood. One contributing factor to this gap in knowledge is the alteration of methylation levels at the CpG site of lncRNAs, as exemplified by C3orf67-AS1 [19]. Further research is necessary to unveil the intricate molecular interactions and signaling pathways involved in the ginsenoside-mediated regulation of lncRNAs.

### 3.1 Transcriptional regulation

In the context of transcriptional regulation, lncRNAs exert their influence over gene expression by interacting with key molecular players such as transcriptional factors and histones [73,74]. The present section also delineates the impact of lncRNAs on genes within a signaling pathway, wherein their expression undergoes modulation. One illustrative instance of this intricate regulatory dance involves the adjustment of histone acetylation, as demonstrated in the study of lncRNA H19. The suppression of H19 resulted in a reduction of osteopontin mRNA and protein levels in Rh2-treated cells, achieved through the inhibition of histones H3 and H4 acetylation on the osteopontin promoter [37]. LncRNAs induced up- or down-regulation of various genes such as PTEN [51], E-cadherin [50], MMP2, MMP9 [53] (Rg3), SOCS3, NRF2 [45] (Rg1), and iNOS [64] (R1).

### 3.2 Sponging microRNAs

The term "sponging activity" refers to a regulatory mechanism by which lncRNAs act as molecular sponges for miRNAs [75]. By binding to miRNAs, lncRNAs prevent them from interacting with their mRNA targets. The sequestration of miRNAs by lncRNAs can result in increased stability and expression of the target mRNAs that would have been otherwise downregulated by the miRNAs. Dysregulation of this sponging activity can have implications in diseases, including cancer, where aberrant expression of lncRNAs and miRNAs is often observed [76,77]. Of the approximately 50 lncRNAs reviewed in the current manuscript, 10 are known to act as miRNA sponges (Table 1, Table 2, Table 3, Table 4). Rg3, for example, has been implicated in upregulating FDFT1 by reducing miR-4425 levels, thereby inhibiting ovarian cancer progression [78]. MiR-4425 has been found to be sponged by the tumor-suppressive lncRNA HCG11 in glioma, indicating a complex interplay between ginsenosides, lncRNAs, and microRNAs [79].

### 3.3 Protein modulation

LncRNA NKILA is upregulated by NFκB and binds strongly to NFκB/IκB. NKILA masks IκB phosphorylation motifs, thereby stabilizing the NFκB/IκB complex by inhibiting IκB phosphorylation and NFκB activation [11]. Rg3 suppresses NKILA accumulation and reverses its stimulation of the Warburg effect and angiogenesis in glioma [52]. H19, when complexed with the methyl-CpG-binding domain protein 1 (MBD1), binds to the control region of its target genes and finely tunes histone methylation [80]. As described previously, the expression of H19 is regulated by various ginsenosides, including Rg3, Rh2, and Rb3.

## 4 Conclusions

In summary, this review explores the intricate relationship between ginsenosides and long non-coding RNAs (lncRNAs) in cancer and other diseases. Ginsenosides like Rh2, Rh3, Rg1, and Rg3 demonstrate significant potential in regulating lncRNAs to hinder cancer progression by inhibiting cell proliferation, inducing apoptosis, and suppressing metastasis. These interactions also hold promise for interventions in cardiovascular disorders, neurodegenerative diseases, inflammation, and metabolic disorders. Highlighting the pivotal role of lncRNAs in mediating ginsenoside-induced anticancer effects, the review discusses specific lncRNAs such as CFAP20DC-AS1, C3orf67-AS1, H19, GAS5, STXBP5-AS1, and RFX3-AS1. Additionally, it explores the potential of using lncRNAs as diagnostic markers and emphasizes the need for further research to comprehend the complete spectrum of ginsenoside-influenced lncRNAs in cancer. Overall, this concise review contributes to our understanding of ginsenoside epigenetics and presents new opportunities for personalized therapeutic interventions in cancer and beyond.

## Conflicts of interests

The authors declare that they have no conflicts of interests.
